# Proportion of HIV exposed infants aged 0–6 months that missed nevirapine prophylaxis in Mulago National Referral Hospital, Uganda: a cross-sectional study

**DOI:** 10.21203/rs.3.rs-2691820/v1

**Published:** 2023-04-05

**Authors:** Nasambu Hellen, Rujumba Joseph, Mupere Ezekiel, Semitala Fred, Musoke Philippa, Ronald Senyonga

**Affiliations:** Makerere University; Makerere University; Makerere University; Makerere University; Makerere University; Makerere University

**Keywords:** HIV exposed, infant, nevirapine prophylaxis

## Abstract

**Background:**

Nevirapine prophylaxis has been found to lower the risk of HIV transmission in breast-fed infants. While about 95% of pregnant and lactating mothers use Antiretroviral therapy in Uganda, a smaller percentage of HIV exposed infants (HEI)receive nevirapine (NVP)prophylaxis. This study aimed to determine the proportion of HEI whomissed NVP prophylaxis and associated factors.

**Methods:**

This was a cross-sectional study done using quantitative methods. It was conducted at Mulago National Referral Hospital. A total of 228mother-infant pairs were enrolled.The proportion of HEI who missed NVP, maternal, infant and health facility factors associated were measured using a pre-tested questionnaire. Bivariate analysis and binary logistic regression model were used to determine the proportion and factors associated with missing NVP prophylaxis.

**Results:**

The proportion of HEI who missed NVP prophylaxis was 50/228(21.9%). Factors significantly associated with HEI missing NVP prophylaxis included; delivery from outside government health facilities [AOR=8.41 95% (CI 3.22–21.99)], mothers; not undergoing PMTCT counselling [AOR=12.01 95% (CI 4.53–31.87)], not on ART[AOR=8.47 95% (CI 2.06–34.88)] and not having disclosed their HIV status to their partners [AOR=2.80 95% (CI 1.13–6.95)].The HEI that missed nevirapine and were HIV positive were 35(70.0%).

**Conclusion:**

One in five HEI missed NVP prophylaxis and nearly three quarters of those who missed NVP prophylaxis were HIV infected. Improving uptake of nevirapine by HEI will require interventions tostrengthen PMTCT counselling, assisted partner notification, reduction of HIV stigma and support to the private sector in the provision of PMTCT services.

## Background

In 2018, the estimated new annual paediatric HIV infection (case rate) was 466 per 100,000 live births, far above the elimination target of < 50 new infections per 100,000 live births, and most of the infections occurred during the breast-feeding period(1).For over two decades, the elimination of new HIV infections among children has been of paramount concern to the global HIV community(2). Recommendations for reducing vertical transmission which accounts for the majority of pediatric infections in low resource settings were updated by WHO to include option B+. This requires all pregnant and breastfeeding HIV-infected women, regardless of CD4 cell count, to continue ART for life known as “option B+” while their infants receive dailyNVP or zidovudine from birth to 4 to 6 weeks(3). Both maternal ART and infant NVP prophylaxis strategies are safe and associated with very low breastfeeding HIV transmission (4). However, the number of HIV Exposed Infants (HEI) who receive ART prophylaxis has stagnated at around 42%(5). A recent study conducted at Mulago Hospital in 2019 studied factors influencing maternal adherence to infant’s NV prophylaxis regimen(6) but did not establish the proportion of HEI that missed nevirapine prophylaxis and factors associated with missing NVP prophylaxis.

We determined the proportion of and factors associated with missing NVP prophylaxis within the first 72 hours of life among HEI managed at Mulago Hospital, a national referral hospital in central Uganda.

## Methods

### Study design

This was a cross-sectional study using quantitative methods of data collection conducted between April 2019 and December 2019.

## Study Setting

The study was conducted at Mulago National Referral Hospital (MNRH), Kampala Uganda[in the immunization clinics and ACU]. Mulago Hospital is Uganda’s National Referral hospital and the teaching hospital for Makerere University College of Health Sciences. It is located the central region, 3km north of Kampala and receives patients from all the suburbs of Kampala and referrals from all over the country. Kampala is the capital city of Uganda. The hospital has a total bed capacity of 1500 in-patient beds, an inpatient turnover of 120,000 patients and attends to over 500,000 outpatients annually.

The Paediatric Department is made up of seven wards, one of them being the emergency ward, Acute Care Unit (ACU).Paediatric patients are received at the Assessment Centre from where they receive outpatient care and those who need specialist care or hospitalization are referred to the ACU. The ACU runs an inpatient section and it admits a minimum of 500 infants and children annually. On average, about 30 HIV exposed infants are admitted per month. It is made up of a resuscitation room that has 3 examination coaches and 1 baby coat, a neonatal room that has 8 baby coats, Paediatric Intensive Care Unit (PICU) that has 6 beds, High Dependency Unit(HDU) and the main ward that have 38 beds and 18 baby coats all together.ACU also has a laboratory where basic tests of Blood slide for malaria and grouping and cross matching are done. ACU admits critically ill children for resuscitation and emergency care over a 24 hour period. In addition to these, there’s a PIHTC point where all mothers admitted at ACU have a mandatory HIV test done after consent and pre-testing counselling is done. Lately, a DNA-PCR point of care machine has been introduced where HIV testing is done for children below 18 months of age and results are got immediately.

The immunisation clinic offers all recommended vaccines as per the Uganda National Expanded Program of Immunization (UNEPI) schedule and also have a Provider initiated HIV testing and counseling (PIHTC) point where all mothers who have not been tested for HIV in a period of a month or more are tested. The clinic runs daily from Monday to Friday from 9 a.m. to 1 p.m. The clinic receives mostly infants and children within the surrounding suburbs of Mulago because most mothers are encouraged to immunize their children from the nearest health facility. On average, 70 infants and children under 5 years of age are immunized and about 10% of these are HIV exposed.

The mother infant pairs were recruited daily from Acute Care Unit and on all clinic days in the immunization clinics.

## Results

A total of 240 mother infant pairs were screened for the study. Seven met the exclusion criteria because the care taker was not the mother and five did not consent. A total of 228 were enrolled.

## Demographic Characteristics

One hundred thirty seven infants were male (57.9%). The mean age of the infants was 4.0 months ± 0.3. Two hundred sixteen were born at term (94.7%) and 63 (27.6%) were hospitalized at birth (Table 1).

Most mothers 127 (55.7%) were aged 26–30. Mothers who were on ART were 209 (91.7%), 126 (55.3%) had disclosed their HIV status to partner were, 75 (32.9%) attended ANC with partner, 191 (83.8%) underwent PMTCT counselling and 184 (80.7%) delivered within a government facility (Table 2).

Factors associated with HEI missing NVP prophylaxis at bivariate analysis are shown in Table 3& Table 4 and at multivariable analysis, delivery from outside government health facilities [AOR = 8.41 95% (CI3.22–21.99)], mothers not undergoing PMTCT counselling [AOR = 12.01 95% (CI 4.53–31.87)], mothers not on ART [AOR = 8.47 95% (CI 2.06–34.88)] and mothers not having disclosed their HIV status to their partners [AOR = 2.80 95% (CI 1.13–6.95)] were significant factors (Table 5).

## Proportion Of Hei Who Missed Nevirapine Prophylaxis

One out of five HEI in the study (21.9%) missed nevirapine prophylaxis within the first 72 hours. This is high given that the study was done at a tertiary hospital with relatively more staff and an established PMTCT program compared to rural health facilities. The facility also receives patients from various regions of the country. It is a reflection of gaps in provision of NVP prophylaxis at birth. This is a hindrance to achieving eMTCT. This is higher than what was observed elsewhere. A retrospective study done in 2013 in 145 health facilities in 24 districts of central Uganda, by Muhumuza et al found that 17.0% of the HIV exposed infants did not receive daily NVP from birth to 6 weeks postpartum(7). This study was done during the early phase of Option B + roll-out in Uganda and could explain the slightly lower percentage of missed NVP compared to our study.

Another retrospective study done in northern Uganda revealed 86.9% HEIreceived NVP prophylaxis from birth until 6-weeks leaving 13.1% who missed NVP prophylaxis(8). This study aimed to find out retention of HEI and associated factors. The percentage could have been lower because they conducted a retrospective study where information could have been missing and it may have been dificult to ascertain the exact numbers.

Between 2002 and 2007, a single dose NVP PMTCT service was implemented in 53 rural villages of southwest Uganda. Twenty-five of them were HIV-surveillance study villages. The surveillance aimed to determine the proportions of mothers testing positive and mother and newborns receiving and ingesting single dose NVP and associated factors. The percentage of HEI that missed NVP prophylaxis within 72 hours was 48%(9). This is higher than what was found in this study because this was a surveillance done in villages and so the likelihood of capturing more HEI that missed NVP prophylaxis is high because some HEI are not brought to hospitals.

In a cross-sectional surveillance study of mother-infant pairs using umbilical cord blood samples collected between June 10, 2007, and October 30, 2008, from 43 randomly selected facilities providing delivery services in Cameroon, Côte d’Ivoire, South Africa, and Zambia, there was random sampling of sites with services to prevent mother-to-child HIV transmission done. A percentage of 49% of HIV-exposed infants received the minimal regimen of single-dose nevirapine (10). Continued missed NVP prophylaxis reflected in these studies hinders eMTCT.

## Factors Associated With Missing Nevirapine Prophylaxis

Mothers who had not undergone PMTCT counselling were 12 times more likely to have HEI missing nevirapine prophylaxis.

Mothers who delivered from outside a health facility were 8.4times more likely to miss nevirapine prophylaxis and hence increased risk of HIV transmission. Delivery within a government health facility increases the chances of an infant receiving nevirapine prophylaxis hence reducing on the possibility of missing NVP(5).

Mothers who were not on ART were 8.5 times more likely to have an HEI that missed nevirapine. The risk of mother-to-child transmission of HIV is higher among women who are not on antiretroviral therapy(12). It is common for women to gradually stop taking ARV drugs after giving birth, which not only compromises their health but also puts their infant’s at an increased risk of acquiring HIV during breastfeeding(13). In a study done in Cameroon, HIV transmission rate differed by maternal prophylaxis:1.7% for HAART, 2.7% for dual therapy and 15.7% for mothers who were not receiving ART with a p-value of < 0.001(14).Taking ART shows that a mother minds about both her and the infant’s health. This explains the correlation between taking ART and infant nevirapine syrup uptake. A study carried out in central Uganda found that among mothers taking ARVs, the levels of maternal adherence to NVP prophylaxis regimens were above 70%. This study did not look at mothers who were not taking ARVs and did not analyse infant NVP uptake(6).Some of our key informants linked missed NVP and ART to misguidance from religious leaders. This is different from what was reported in other studies. A descriptive study carried out to examine the relationship between religiosity and ART adherence in a sample of 220 patients attending an HIV/AIDS clinic in a Ugandan public hospital showed that high religiosity was associated with high adherence to ARV drugs(15).

### HIV infection among HEI who missed NVP.

Almost three quarters of HEI who missed NVP prophylaxis were HIV infected. In this study, there was a 70% risk of HIV positivity among HEI that missed NVP prophylaxis. With the proven efficacy of nevirapine, HIV infection is lower in infants who take nevirapine compared to those who do not. The SWEN study showed that once-daily infant nevirapine for 6 weeks resulted in a 53% reduction in postnatal HIV infection at age 6 weeks (46).A study done by Kahungu et al found that HEI who did not receive ART prophylaxis at birth were five times more likely to be HIV infected than those who received prophylaxis(19).

This study also found that 5 out of 40(12.5%) HEI were HIV infected despite receiving nevirapine prophylaxis. This is in sync with the fact that undetectable viral load in a mother and infant prophylaxis does not equate to non-transmission of HIV virus to the infant(13).

## Conclusion

One in five HEI in this study missed their nevirapine prophylaxis. Factors significantly associated with infants missing nevirapine prophylaxis were; mothers not undergoing PMTCT counselling, mothers not on ARVs, non- disclosure of HIV status to their partners and delivering from outside a government health facility. Nearly three quarters of HEI who missed NVP were HIV infected. There should be more emphasis oninfant NVP prophylaxis through interventions that strengthen PMTCT counselling, assisted partner notification, reduction of HIV stigmaand support to the private sector in the provision of PMTCT services.

## Figures and Tables

**Table 1:** showing demographic characteristics of HIV exposed infants aged 0–6 months at MNRH.

Variable	HEI who received NVP n = 178(%)	HEI who missed NVP n = 50(%)	Total N = 228
Age in months			
< 1 month	22(75.9%)	7(24.1%)	29
1 to <2	19(86.4%)	3(13.6%)	22
2 to <3	21(72.4%)	8(27.6%)	29
3 to <4	25(75.8%)	8(24.2%)	33
4 to <5	20(80.0%)	5(20.0%)	25
5 to <6	71(78.9%)	19(21.1%)	90
Gender			
Male	101(76.5%)	31(23.5%)	132
Female	77(80.2%)	19(19.8%)	96
Gestational age at birth			
Term	171(79.2%)	45(20.8%)	216
Preterm	7(58.3%)	5(41.7%)	12
Delivery mode			
Vaginal	155(76.4%)	48(23.6%)	203
Caeserean section	23(92.0%)	2(8.0%)	25
Hospitalisation after birth			
Yes	31(49.2%)	32(50.8%)	63
No	78(47.3%)	87(52.7%)	165

**Table 2:** showing demographic maternal characteristics

Variable	HEI who received NVP n = 178(%)	HEI who missed NVP n = 50(%)	Total N = 228
Age in years			
<=19	8(66.7%)	4(33.3%)	12
20–25	49(76.6%)	15(23.4%)	64
26–30	97(76.4%)	30(23.6%)	127
>30	24(96.0%)	1(4.0%)	25
Taking ARVs			
Yes	173(82.8%)	36(17.2%)	209
Disclosure of HIV status to partner			
Yes	112(88.9%)	14(11.1%)	126
Attended ANC with partner			
Yes	69(92.0%)	6(8.0%)	75
Underwent PMTCT counselling			
Yes	168(88.0%)	23(12.0%)	191
When was the 1 st ANC visit			
1st trimester	44(9.6%)	3(6.4%)	47
2nd trimester	107(79.9%)	27(20.2%)	134
3rd trimester/no visit	27(62.8%)	20(37.2%)	43
Place of delivery			
Gov’t facility	157(85.3%)	27(14.7%)	184
Non gov’t facility	21 (47.7%)	23(52.3%)	44
Household income			
> 200,000/=($50)	49(68.1%)	23(31.9%)	72
200,000/=($50)–50,000/=($15)	101(82.8%)	21(17.2%)	122
< 50,000/=($15)	28(82.4%)	6(17.6%)	34

PMTCT: Prevention of mother to child transmission Gov’t: Government

TBA: Traditional birth attendant ARVS: Antiretrovirals ANC: Antenatal care

**Table 3:** showing bivariate analysis of infant factors associated with HEI missing NVP prophylaxis

Variable	Number x/n (50/228)	Percentage (%)	Unadjusted Odds ratio (95% CI)	p-value
Age in months				
< 1 month	7/29	24.1	1.00	
1 to <2	3/22	13.6	0.50(0.11–2.20)	0.356
2 to <3	8/29	27.6	1.20(0.37–3.90)	0.765
3 to <4	8/33	24.2	1.01(0.31–3.23)	0.992
4 to <5	5/25	20.0	0.79(0.21–2.88)	0.716
5 to <6	19/90	21.1	0.84(0.31–2.27)	0.732
Sex				
Male	31/132	23.5	1.00	
Female	19/96	19.8	0.80(0.42–1.53)	0.507
Mode of delivery				
SVD	48/203	23.7	1.00	
C/S	2/25	8.0	0.28(0.06–1.24)	0.093
Gestation age				
Term	45/216	20.8	1.00	
Preterm	5/12	41.7	2.71(0.82–8.98)	0.102
Hospitalisation at birth				
No	37/165	22.4	1.00	
Yes	13/63	20.6	0.90(0.44–1.84)	0.771

**Table 4:** showing bivariate analysis of maternal factors associated with HEI missing nevirapine prophylaxis

Variable	Number x/n (50/228)	Percentage (%) (row percentage)	Unadjusted Odds ratio (95% CI)	p-value
Age in years				
</= 19	**4/25**	**33.3%**	**1.00**	
20–25	**15/64**	**23.4%**	**0.61(0.16–2.33)**	**0.471**
26–30	**30/127**	**23.6%**	**0.62(0.17–2.20)**	**0.459**
>30	**1/25**	**4.0%**	**0.08(0.01–0.86)**	**0.037**
Taking ARVs				
Yes	**36/209**	**17.2%**	**1.00**	
No	**14/19**	**73.7%**	**13.46(4.55–39.81)**	**0.001**
Duration of taking ARVs				
< 3 months	**16/23**	**69.6%**	**1.00**	
12 months	**8/47**	**17.0%**	**0.09(0.03–0.29)**	**0.001**
>1 year	**12/139**	**8.63%**	**0.04(0.01–0.12)**	**0.001**
Disclosure of status to partner				
Yes	**14/126**	**11.1%**	**1.00**	
No	**36/102**	**35.3%**	**4.36(2.19–8.70)**	**0.001**
Attending ANC with partner				
Yes	**6/75**	**8.0%**	**1.00**	
No	**44/153**	**28.8%**	**4.64(1.87–11.50)**	**0.001**
Underwent PMTCT counselling				
Yes	**23/191**	**12.0%**	**1.00**	
No	**27/37**	**73.0%**	**19.72(8.44–46.06)**	**0.001**
First ANC visit				
1st trimester	**3/47**	**6.4%**	**1.00**	
2nd trimester	**27/134**	**20.2%**	**3.70(1.06–12.87)**	**0.040**
3rd trimester and no visit	**20/47**	**42.55%**	**10.86(2.94–40.17)**	**0.001**
Place of delivery				
Gov’t facility	**27/184**	**14.7%**	**1.00**	
Non gov’t facility	**23/44**	**52.3%**	**6.37(3.10–13.09)**	**0.001**

Gov’t: GovernmentTBA: Traditional birth attendant

ARVS: Anti-retroviralsANC: Antenatal care

PMTCT: Prevention of mother to child transmission

**Table 5:** showing multivariate analysis of factors associated with missing NVP prophylaxis

Variable	Adjusted Odds ratio (95% CI)	p-value
PMTCT counselling		
Yes	1.00	
No	12.01(4.53–31.87)	0.001
Place of delivery		
Gov’t facility	1.00	
Non gov’t facility	8.41(3.22–21.99)	0.001
Taking ARVs		
Yes	1.00	
No	8.47(2.06–34.88)	0.003
Disclosure of status to partner		
Yes	1.00	
No	2.80(1.13–6.95)	0.026
First ANC visit		
1st trimester	1.00	
2nd trimester	2.84(0.70–11.48)	0.142
3rd trimester and no visit	2.76(0.61–12.42)	0.185

Gov’t: Government PMTCT: Prevention of mother to child transmission ARVS: Anti-retrovirals ANC: Antenatal care

## Data Availability

The datasets used and/or analysed during the current study are available from the corresponding author on reasonable request.

## Abbreviations

ART: Anti-retroviral therapy
HEI: HIV exposed infant
KII: Key Informant Interview
MoH: Ministry of Health
MNRH: Mulago National Referral Hospital
MTCT: Mother to Child Transmission
NVP: Nevirapine
PCR: Polymerase Chain Reaction
PMTCT: Prevention of Mother to Child Transmission
